# A Qualitative Study of Men’s Experiences Using Navigate: A Localized Prostate Cancer Treatment Decision Aid

**DOI:** 10.1177/23814683231198003

**Published:** 2023-09-13

**Authors:** Elizabeth Todio, Penelope Schofield, Jessica Sharp

**Affiliations:** Department of Psychological Sciences, Swinburne University of Technology, Melbourne, VIC, Australia; Department of Psychological Sciences, Swinburne University of Technology, Melbourne, VIC, Australia; Iverson Health Innovation Research Institute, Swinburne University, Melbourne, VIC, Australia; Behavioural Sciences Unit, Health Services Research and Implementation Sciences, Peter MacCallum Cancer Centre, Melbourne, VIC, Australia; Sir Peter MacCallum Department of Oncology, The University of Melbourne, Parkville, VIC, Australia; Department of Psychological Sciences, Swinburne University of Technology, Melbourne, VIC, Australia

**Keywords:** decision aid, localized prostate cancer, patient preferences, qualitative interview, treatment decision

## Abstract

**Background.** Men diagnosed with localized prostate cancer (LPC) often face a dilemma in choosing between available treatment options that have similar survival rates but for which the perceived advantages and disadvantages of each treatment differ. The Navigate decision aid was created to assist Australian men with LPC in making informed decisions about treatment that align with their personal values and preferences. Navigate presents current, unbiased information, including an interactive values clarification exercise. **Objective.** This study was a qualitative investigation of men’s treatment decision making for LPC, and their experiences using the Navigate Web site, to identify areas for improvement and inform implementation. **Methods.** Semi-structured interviews were conducted with 20 men diagnosed with LPC who completed the intervention arm of the Navigate randomized controlled trial. Interview transcripts were thematically analyzed. **Results:** Five main themes emerged: 1) diagnosis experiences varied, although men were strongly influenced by their clinician to make an early initial treatment decision; 2) men sought resources and support they trusted; 3) men valued Navigate’s multiformatted content and design; 4) men suggested more content was needed on a) the diagnosis journey and b) new treatment updates; and 5) men identified design flaws in the values clarification exercise on Navigate but appreciated the tool being available. **Conclusions.** Specialist authority influenced men to make an early treatment decision. However, Navigate was helpful in supporting men’s ongoing treatment decision making, particularly men on active surveillance who may face further treatment decisions if their cancer progresses. To gain trust and improve engagement from Navigate users, credentials and sources of information need to be prominent. Trustworthiness, timing of access, and the clinician’s role in empowering men to use available decision aids are crucial elements to be considered when implementing Navigate in clinical settings.

**Highlights:**

- The Navigate decision aid Web site was created to help Australian men diagnosed with localized prostate cancer (LPC) make an informed decision about their treatment.
- Navigate was helpful in supporting men’s ongoing treatment decision making for LPC.
- Men’s treatment decision making for LPC was greatly influenced by perceived authority and trust in their clinician.
- Trustworthiness, timing of access, and the clinician’s role in empowering men to use available decision aids are crucial.

## Graphical abstract

**Graphical Abstract:**
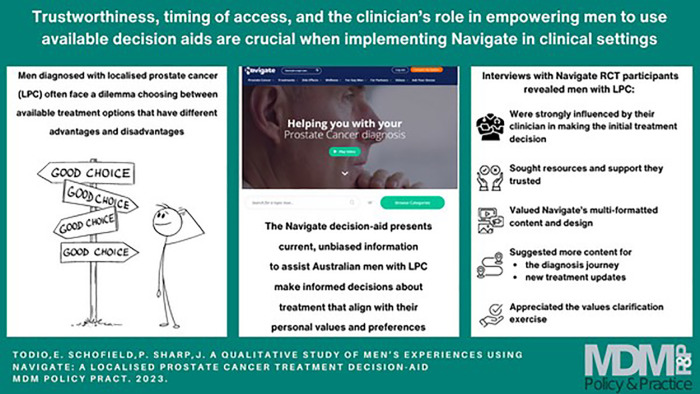
This is a visual representation of the abstract.

Prostate cancer is the most commonly occurring cancer among men globally, where it is estimated that more than 1.4 million new cancers were located in the prostate in 2020.^1^ Patients diagnosed with prostate cancer are also reported to have one of the highest survival rates compared to any other cancer type in most high-income countries,^2^ with the notable increase in prostate cancer incidence attributed to the rise in diagnosis testing in various developed countries.^1^ Consequently, cases of prostate cancer are more likely to be low-grade and localized prostate cancer (LPC).^3,4^ Men diagnosed with LPC often find it difficult to make a treatment and management decision due to the multiple options available, where there is no optimal choice and the fact that personal preferences and lifestyle will influence the decision.^5,6^

Curative treatments (i.e., radical prostatectomy, external beam radiotherapy, brachytherapy) have high survival outcomes, yet these procedures are highly invasive and can have ongoing and distressing side effects, including urinary and bowel incontinence as well as erectile and sexual dysfunction.^7,8^ An alternative management strategy for LPC is active surveillance (AS), which involves regularly monitoring the tumor’s progress while delaying or avoiding radical treatments.^9,10^ Despite AS having equivalent survival outcomes to curative treatments,^7^ some men who are eligible for AS report feeling anxious or confused about not receiving curative treatment.^9,11^ Consequently, men with LPC commonly face ongoing decision-related distress as they need to reflect on the possible side effects of treatment while also considering the risk that their cancer might worsen.^12^ Thus, supporting patients with LPC to make well-informed treatment decisions that align with their personal values and preferences is an important starting point for promoting psychological adjustment and reducing distress.

Decision aids (DAs) have been developed to guide patients through the deliberative task of weighing up the evidence-based pros and cons of each available treatment option while also considering the patient’s preferences and life circumstances.^13^ While there have been several DAs specifically designed for prostate cancer care, systematic reviews have highlighted how many previous studies assessing prostate cancer treatment DAs inadequately address the needs of LPC patients.^5,14^ For instance, older studies examined DAs that included outdated therapeutic options. In addition, some DAs lacked theoretical framework and sufficient systematic evaluation, with notable variations in the instruments used to assess outcomes likely accounting for the inconsistent findings among various decisional outcomes. See a recent review examining the effectiveness and implementation of patient treatment DA tools for men with localized prostate cancer for further evaluation.^14^

The Navigate DA Web site was created to assist Australian men with LPC make informed decisions about their treatment.^15^ Navigate was co-designed by a multidisciplinary team and consumers, is theoretically driven, and complies with the International Patient Decision Aid Standards (IPDAS).^13^ It presents current, unbiased information in various formats including written articles, graphical tables, video presentations of men’s treatment decision-making processes, and their experiences relating to their chosen treatment (e.g., side effects) as well as an interactive values clarification exercise (VCE) to help men understand how their preferences and values align with the treatment options (see Supplementary Material).

The impact of Navigate on patient-reported outcomes, including selected treatment, men’s preparedness for decision making, decisional conflict, regret, and satisfaction, was assessed using a randomized controlled trial (RCT) (to be reported elsewhere).^15^ Men were eligible to participate in the trial if they were recently diagnosed with LPC, had yet to make a treatment decision, and were deemed eligible for AS by their treating clinician. When evaluating patient interventions, it is important that patient experiences using the resource are documented in addition to patient-reported outcomes to understand the complexity of the intervention in the social context.^16^ Qualitative analysis may provide additional evidence as to the strengths and limitations of the DA and therefore valuable insight into optimal ways to implement the DA into the treatment decision-making pathway that might not be gleaned from quantitative methods alone.^14^ Therefore, to complement the Navigate trial,^15^ this study was a qualitative investigation of men’s treatment decision-making experiences for LPC, and their experiences using the Navigate website, to identify specific areas for improvement and inform implementation.

## Method

### Design

A qualitative descriptive design was used with a modified grounded theory approach to allow for the revision of the direction of research as new information emerged.^17,18^ This included grounded theory techniques involving inductive, cyclic, and comparative data evaluation. Research procedures and reporting followed the Standards for Reporting Qualitative Research (SRQR) checklist.^19^

### Ethics and Funding

This study was approved by the Peter MacCallum Cancer Centre ethics committee (HREC/74924/PMCC). The study had no external funding.

### Setting and Participants

The study involved 20 Australian men previously recruited to the Navigate RCT.^15^ Men were eligible to participate in this qualitative study if they completed the Navigate trial and had been randomized to the intervention arm (i.e., access to the Navigate DA Web site). A purposive sampling technique was used to recruit participants from different states across Australia and from metropolitan and regional/rural areas. Patients were recruited from 1) the Peter MacCallum Cancer Centre site, 2) 5 clinicians across Queensland and Victoria who directly referred their patients, and 3) self-referred men who expressed their interest through the Navigate website. Participants were considered appropriate for the study as they were patient consumers of the Navigate decision aid Web site and therefore ideal to provide insight into decision making and user experience.^20^

### Procedure and Materials

Interviewing and recruitment for this study were undertaken by E.T., a research assistant of the Navigate RCT who had recruited and consented men into the trial. E.T. was provided training with an external expert qualitative researcher to ensure competent qualitative data analysis methods were used. E.T. accessed the Navigate trial participant list and worked backward by approaching men who most recently exited the trial. Men were sent a study invitation e-mail that included an attachment of the participant information sheet. This was followed by a phone call from E.T., who explained the study and evaluated their willingness to participate. There was no funding for this study, and eligible men were invited to participate voluntarily. For those who expressed interest, an interview time was scheduled via phone or videoconferencing depending on preference. Interviews were conducted between July–August 2021 and February–April 2022.

All participants were sent login details to Navigate prior to the interview to allow them to review the content of the Web site. Informed consent was received verbally and audio-recorded at commencement of the interview. During the interview, E.T. guided the participant through the different areas of the Navigate Web site while asking specific questions related to the Web site. A purpose-designed interview schedule specifically developed for this study (see Supplementary Material) included basic demographic questions followed by semistructured questions related to men’s treatment decision experiences, Web site design and usability, user engagement, thoughts about specific elements of Navigate (e.g., articles, videos, values clarification exercise), and their recommendations for implementation.

### Data Analysis

Audio-recorded interviews were transcribed verbatim by an external transcriber throughout the data collection phase. E.T. reviewed the transcribed interviews against the audio recordings to ensure accuracy and removed identifiable information from the transcripts. Atlas.ti9, a qualitative data management software, was used by the researchers to analyze the transcribed interviews (develop code book, formulate categories, identify themes). E.T. initially coded the interviews by labeling text segments that were relevant to the research aims.^21,22^ To ensure rigor,^23^ an interrater reliability strategy was implemented with J.S., a PhD qualified researcher experienced in qualitative methods, who is unaffiliated with the Navigate trial. J.S. independently analyzed the transcripts and reviewed the initial coding to verify the analysis. E.T. and J.S. then collated comparable codes into specific categories. Themes were developed to represent similar groups of categories, whereby the 2 researchers continued to discuss and adapt interpretations of the codes and categories until reaching agreement on the themes. The codes, categories, and themes were reviewed for accuracy and relevance by P.S., the lead researcher of the Navigate trial and renowned psycho-oncology researcher with more than 30 y of experience conducting qualitative research. Acknowledging that meaning is generated from data and judgments about saturation are subjective,^24^ the researchers determined that 20 patient interviews were adequate once the data collected met the aims of the study and no new themes had been gleaned from continued analysis.^25^

## Results

Of the 33 men approached for interview, 8 men declined and 5 men did not respond to the study invitation. Twenty men gave their informed consent and completed the interview. Characteristics of these participants who consented to the study are shown in Table 1. Most men chose AS to manage their low-risk prostate cancer, many resided in a major city, most had private health insurance, and almost all had a partner. The average interview time was 50 min (range: 29–137 min). The duration from when participants completed the Navigate trial to when they were interviewed ranged from 7 d to 21 mo.

**Table 1:** Characteristics of Participants^a^

Characteristic	Participants (*N* = 20)
Age, y	*x̄* = 65.65 (*s* = 8.52; range 49–77)
State	
ACT	2 (10.0)
NSW	2 (10.0)
QLD	7 (35.0)
VIC	9 (45.0)
Geographic area	
Metro	13 (65.0)
Regional/rural	7 (35.0)
Current chosen treatment	
Active surveillance	14 (70.0)
Brachytherapy	2 (10.0)
External beam radiotherapy	1 (5.0)
NanoKnife	1 (5.0)
Radical prostatectomy	2 (10.0)
Relationship status	
Married/de facto	19 (95.0)
Widowed	1 (5.0)
Patient type	
Private	16 (80.0)
Public	4 (20.0)
Interview medium	
Phone	12 (60.0)
Videoconferencing	8 (40.0)

a Data are presented as mean (standard deviation; minimum–maximum) or number (%).

Five major themes were identified from the data. Men’s recommendations were also deductively extracted from interview transcripts. Table 2 presents examples of participants’ statements that informed the themes and categories, and illustrative quotes are also presented in the following sections. Contextual information is provided with the first quote of each participant.

**Table 2:** Textual Examples Informing Themes and Categories Depicting Men’s Experiences with the Navigate Web Site

Themes and Categories	Text Illustrations
**Theme 1: Diagnosis experiences varied, but all men decided on a treatment early**	
1.1 Clinician’s influence on men’s decision making	(a) “. . . he calls me in, about a 3 week wait or so, sit down, ‘Yes I can confirm that you do have cancer.’ . . . And within 30 seconds of that statement, he has now got his laptop in my face showing me a movie of him doing a radical proctectomy by robotic surgery on some unfortunate being. . . . I’m still wrestling with the fact that I’ve got cancer. He says, ‘Well this is what I recommend. This is the gold standard. And if you’re okay you can come in next week and we can get it done.’ No options. That was it. I was stunned, absolutely stunned.” (P5, age 68 y, partner, metro) (b) “The first doctor said he wanted to have me on the table within 4 weeks to get the prostate out. . . . But the way he spoke too, he tried to steer me in the direction of what he called the ‘gold standard,’ the removal of the prostate. I had to talk to him round about not wanting an operation and preferring to have some sort of radiation treatment, instead of the operation. Then he did recommend me to go to a radiation doctor who he knew, and deal with, and that was really good. I chose that path, and I am very happy I did.” (P4, age 62 y, partner, metro) “At first, when I first saw my specialist, he told me I had no other option than to have surgery and all this, until I got on to Peter Mac. Then, they put me on to just monitoring it because it wasn’t as bad as it was made out [to be].” (P10, age 49 y, partner, metro) (c) “[My doctor] had told me that the others weren’t really options, so that was an easy solution for me. Especially when he said, ‘This has grown dramatically, we need to act sooner not later.’” (P2, age 67 y, partner, metro)
1.2 Men made an early initial treatment decision	(a) “I’m not the type of person that sits around and does nothing.” (P14, age 75 y, no partner, metro) “Once I had made my decision to go ahead with the surgery . . . I was convinced that that was just something that I had to do, and I wasn’t interested in looking at any of the other treatments that didn’t involve the removal of the prostate. I just said, no look that’s the way I’m going. Booked it in and I never looked back really. I was just working toward that surgery date and that’s what’s actually happened.” (P8, age 57 y, partner, metro)
1.3 Men varied with how involved they were in their treatment decision depending on their PC risk	(a) “I’ve got other things to do, so at the end of the day I’m not really looking for further information at this point.” (P16, age 57, partner, regional/rural) “Obviously if it was not great news I would be seeking out as much information as I can.” (P20, age 80, partner, metro) (b) “I want to be an active agent in the management of my cancer” (P18, age 59 partner, metro).
**Theme 2: Trustworthiness was an important factor in seeking resources and support**	
2.1 Men were sceptical using the internet as an information source	(a) “All I was doing is going through my phone, putting in prostate cancer treatments and that kind of stuff and getting all kinds of information from all of the world actually. It wasn’t all that clear, especially the information I was getting around life expectancy and that sort of stuff, was pretty dire really.” (P3, age 70 y, partner, metro) (b) “That was my dilemma when I was going over the Web before, picking bits and pieces out of everywhere, how do I now rate those as peer-review?” (P5)
2.2 Improving credibility in the Navigate website	(a) “I think that what this Web site needs to be approved and certified by specialists. That it is the premier Web site for people to use and the Australian Medical Association should be involved. Saying that for prostate cancer this is the way to go.” (P1, age 77 y, partner, metro)
2.3 Social support played a significant role in the decision-making process	(a) “There are three blokes I know of who’ve had radical [prostatectomy], so it’s obviously a very common condition. And their experiences have sort of helped me come to terms with my own situation.” (P9, age 66 y, partner, regional/rural) “The best resources for me was going to . . . the prostate cancer meeting. . . . A group of guys that get together once a month and discuss their experiences, what they’ve been through, their results, how they’re feeling.” (P4) (b) “And, I got, my wife also, she got access to it [Navigate] and she was able to work through the bits that were relevant to herself. And we were able to sit down together and discuss it so . . . it is excellent.” (P9) “I think it’s fantastic. And I’ll say it [Navigate] was really useful for my wife and I.” (P2)
**Theme 3: Men’s feedback on Web site design and graphic and video content was generally positive**	
3.1 Men were satisfied with Web site design and usability	(a) “I think it was user-friendly and it was easy to find all of the information you’re after, and logically set out. So, I certainly didn’t have any issues with the design or layout at all.” (P8) “Yeah, I loved it. I just found it was so helpful, easy to navigate. I suppose that’s why it’s called Navigate. Easy to work your way through the various dropdown menus and find the information I felt I needed to find.” (P9) (b) “I didn’t go into this blind and Navigate’s not the only Web site, but I think it puts together everything very well . . . no other place is put together quite like this, so it’s good.” (P1) “I think as a resource, it’s something I couldn’t find when I needed it. When I went to various [Web sites] from the States and the rest of it. . . . I was going around, finding something out on this site, something else on that site, something somewhere else. . . . I think Navigate does a great job. . . . It solves that puzzle.” (P5)
3.2 Navigate videos of patient experiences were valuable in confirming men’s chosen decision	(a) “I thought it was well put together and probably the video interviews were some of the most helpful things. You know, people recounting their experiences of going through the same sorts of things that I’ve now had and been through myself.” (P8) “What I found most useful was the videos. Hearing individuals talking about their own experiences, talk about why they made decisions, what happened when they had surgery, what happened when they had their treatments, how they chose. One guy I think he had difficulty about how he made decisions. I think that was useful, and I found the video system was really cool.” (P2)
3.3 Treatment comparison table was useful, but men would have preferred data sources to be more prominent	(a) “I like table layouts because it’s clear isn’t it? You know you just go down your cross the table and you go there’s this this or this. There’s brachytherapy and this means that. Maybe I don’t want to have high-energy X-rays. It makes it easy to determine what you want to do or how to review your options.” (P2) “It’s to the point, short answers which is good. Gives your options you can pick from. Very good.” (P6, age 66 y, partner, regional/rural) (b) “I suppose I’m interested in where the data comes from—is it really 9 out of 10 men?” (P2) “How true is that? I don’t know. . . . To me the information I got from overseas Web sites, and then looking at that 5 out of a 100 is way underestimating the risk.” (P3) “The place I saw probabilities was up there 9 out of 10. That’s the only place I think I’ve seen probabilities. I think it could do with a bit more probabilities throughout it, from the point of view of risk. But I don’t know how you do that, and you really need to talk to a medical expert in relation to that. Because I’m not quite sure whether or not the probabilities are known or can be expressed simply.” (P1) (c) “It might even be best to put the words up there and have low risk, ‘less than 100.’ Put the words in, even though it repeats itself. For example, low risk, less than 5 in 100 patients.” (P1) (d) “Maybe just a statement saying the data that is used in this table on this Web site comes from research conducted by Peter Mac cancer centre or whatever . . . because it’s an acknowledgment of who . . . and it’s just because I’m a questioning mind—‘Where does that data come from? You could make it up.’” (P2)
**Theme 4: Navigate articles were an important source of information, but men highlighted areas for improvement**	
4.1 Written content was useful and easy to read	(a) “I actually liked it, that it wasn’t going into too technical terms. That it was in more layman’s terms, I could understand what they were talking about rather than thinking, ‘what the hell are they talking about?’” (P10)
4.2 Men wanted more information about the diagnosis process	(a) “When it comes to the Navigate site, I’ve got a few issues . . . around not covering the diagnosis process and the options available before you have a biopsy.” (P18) “At first glance I haven’t seen any information about biopsies . . . I think that’s an area that needs a bit of thought” (P15, age 74 y, partner, metro) “I ended up having to stay overnight in the hospital that night [after the biopsy], when you normally just . . . go home, but I couldn’t do that. I just wanted to find out a little more about that.” (P4) (b) “Using terminology such as ‘You’ve returned a Gleason score of 7, a 3 plus 4, so you’re regarded as being an intermediate risk category,’ and the material on the Navigate website deals with people who are like you . . . who normally would be looking at treatment options involving various radiotherapy and brachytherapy . . . as well as surgery.” (P8) “That should say, ‘If your Gleason score is X, go to this part.’” (P14) “I think if you could get on and actually read something about Gleason score 7. Something explaining it more clearly.” (P3)
4.3 Some men thought that the Web site was limited in presenting the different treatment options available	(a) “Most people would be quite interested in those other treatments . . . in time you’d like to see some more updates on those” (P13, age 50 y, partner, regional/rural) “I’d probably like to know . . .what the latest machines are, they can change pretty quick.” (P17, age 59 y, partner, regional/rural)
**Theme 5: Men identified design flaws in the VCE but appreciated the tool being available**	
5.1 Men had usability issues with the VCE	(a) “This is why I had difficulty with this. So, before we were looking at killing cancer cells now, we’re actually cutting out, which is a very evocative description. Removing—little softer . . . I feel that I’m getting biased, I’m possibly being biased by the words. No one likes the feeling having something cut out. Would remove to be too softer term? Of course, if you said remove then what’s the difference between question 3 and question 2?” (P5) (b) “You could have a little disclaimer when you get to a question—‘anything that’s in orange provides you more detail.’” (P2) “I hadn’t noticed that light green colour, so if you had it in the orange like you had up the top or you had it in red it’d stand out more.” (P4) “. . . Make it larger writing. The guys that are reading it, their eyes aren’t the best and if you got a colour that doesn’t stand out, it will just blend in.” (P17)
5.2 VCE responses were mixed	(a) “It reinforces that I’d come to right the decision . . . the more I use a tool like that the more I feel comfortable with what I’m doing.” (P12, age 69 y, partner, metro) (b) “It was interesting, I tried to do it as honestly as I could. I did it three times and I couldn’t get to brachytherapy.” (P5)
5.3 Men’s overall impressions of the VCE were positive	(a) “I don’t think there was any time where I thought ‘I’m not sure why they’re asking about this.’ I could understand why the questions were being asked, and I certainly didn’t have a problem in understanding the nature of the questions or the information they were trying to get, so it was all pretty logical and easy to use.” (P8) (b) “I like the confrontational sort of view of it. But it’s really, it’s putting it on the line, isn’t it?” (P2)

PC, prostate cancer; VCE, values clarification exercise.

### Theme 1: Diagnosis Experiences Varied, But All Men Decided on a Treatment Early

#### Clinician’s influence on men’s decision making

Men spoke extensively about their diagnosis experiences, revealing diverse encounters with their clinician. Some men revealed they had complete trust in their clinician’s decision and were willing to go along with whatever their clinician suggested: “I had made up my mind as soon as I’d found out that I’d be guided by my doctor.” (P7, age 74 y, partner, metro). However, several men indicated that they were not satisfied with their initial clinician’s recommended treatment and were not given any other options to consider. This was particularly evident among specialists who were encouraging their patients to go ahead with a surgical procedure (see Table 2, 1.1a). One man expressed that his specialist “was more working on his own, rather than for the patient” (P2). Consequently, some men ended up seeking a second opinion from a different specialist who offered a treatment that more aligned with their preferences (see Table 2, 1.1b). Some men’s diagnosis had developed to a higher risk PC around the time they were given access to Navigate. Therefore, AS was no longer an option given to them by their clinician, where they had to choose a curative treatment (see Table 2, 1.1c).

#### Men made an early initial treatment decision

Men revealed that they had decided on their initial treatment soon after they had been diagnosed. This was apparent among men who were diagnosed with low-risk PC (i.e., Gleason Score 3 + 3 = 6) who all initially chose AS and men with intermediate-risk PC (i.e., Gleason Score 3 + 4 = 7) who decided quickly on a curative treatment option (see Table 2, 1.2a). One man expressed that he did not use Navigate extensively due to having made his decision early on: “[Navigate] didn’t assist me in making a decision, because I already made [my decision]” (P1). However, other men reported they had used Navigate to confirm the decision they had already made, and it played an important role in clarifying their decision: “It reinforced that I’d come to right the decision” (P12).

#### Men varied with how involved they were in their treatment decision depending on their PC risk

Men expressed different levels of interest in understanding the alternative treatments available. Men who were on AS indicated they were “comfortable” sticking to their current management plan rather than dwelling on what curative treatment they may have to choose in the future: “I’ll be making that decision when I have to make it. I am not trying to pre-guess what I would be like by that stage” (P19, age 68 y, partner, regional/rural). Consequently, some men felt they did not need to look at Navigate to review any of the other options once they made their initial decision (see Table 2, 1.3a). In contrast, several men diagnosed with intermediate-risk PC indicated they played an active role in understanding their diagnosis and the types of treatments available: “I was still considering a whole range of different possibilities, so I was actively looking to see what the various options involved” (P8) (see Table 2, 1.3b).

### Theme 2: Trustworthiness Was an Important Factor in Seeking Resources and Support

#### Men were skeptical of using the Internet as an information source

Before having access to Navigate, the most used resource to seek information about LPC diagnosis and different treatments was the Internet: “Basically, I just got on the net and started looking up stuff, because I was so agitated about the fact that there was something wrong with me, for a start” (P3). Some men reported that the different Web sites they used were confusing due to the information not being specific to Australia: “A lot of that stuff is American based. . . . They even talk in different medical jargon” (P2) (see Table 2, 2.1a). Men also recognized that not all information they accessed on the Internet was accurate and questioned the reputation of the Web sites they used: “it got to the point where there was so much information on [the Internet] that wasn’t factual” (P13) (see Table 2, 2.1b). Consequently, men relied on gaining additional information from their specialist whom they trusted: “It was from the specialist I got the resources from essentially . . . and he was very good” (P1).

#### Improving credibility in the Navigate Web site

The men felt that they could trust Navigate because it was made by a reputable organization, although several suggested more emphasis was needed to show that it was created specifically for Australian men: “it would be handy if it had next to the Navigate website, like ‘Australia’ . . . so you know you’re on a trusted site” (P13). Furthermore, men suggested Navigate should demonstrate endorsements from specialists to improve trustworthiness (see Table 2, 2.2a). One man suggested that this was important so that users do not see the Navigate Web site as “another piece of junk mail” (P5).

#### Social support played a significant role in the decision-making process

Several men indicated they spoke to other men they personally knew and trusted to learn about the different treatments to help with their decision making. Men specified that social support from their friends or PC support groups were valuable in helping come to terms with their diagnosis (see Table 2, 2.3a). Some men expressed that it was important to involve their partner when deciding on a treatment, where one man highlighted that it was a “journey” they went through together (P5). The same men revealed that Navigate was a valuable resource that both they and their partner could use to discuss their diagnosis together (see Table 2, 2.3b).

### Theme 3: Men’s Feedback on Web Site Design and Graphic and Video Content Was Generally Positive

#### Men were satisfied with Web site design and usability

Navigate was described by all as user friendly and easy to access the information they wanted (see Table 2, 3.1a). Men particularly liked that the information was all in one place, in comparison to previous information resources on the Internet that they had to mentally compile (see Table 2, 3.1b).

#### Videos of patient experiences were valuable in confirming men’s chosen decision

Most men reported that the videos were the most helpful resource on Navigate, where they were able to hear about other men’s experiences with different treatments chosen and what to expect. Men expressed that they appreciated the honesty of the men in the videos, which helped reinforce their confidence in their chosen decision (see Table 2, 3.2a). In addition, one participant expressed that they were “a good visual aid . . . rather than just having to read” (P10).

#### Treatment comparison table was useful, but men would have preferred data sources to be more prominent

The graphical table comparing the 4 main treatments (see Supplementary Material) was identified as one of the most useful resources on Navigate. Men particularly liked how all the treatments were summarized in 1 table, from which they could easily weigh up the different aspects to consider (see Table 2, 3.3a). However, several men thought the sources of statistical information needed to be highlighted more clearly in the table (see Table 2, 3.3b). One man suggested tweaking the table design to make the specific risk references more prominent (see Table 2, 3.3c). Another advised that it would be important to highlight where the data come from and to make it clear to users that the information originates from a reputable source to reinforce confidence in the data (see Table 2, 3.3d).

### Theme 4: Navigate Articles Were an Important Source of Information But Men Highlighted Areas for Improvement

#### Written content was useful and easy to read

Men generally gave positive comments about the articles on the Navigate Web site, stating that the information was clear and easy to understand: “The articles are brilliant. They’re couched in language that is able to be understood by just about anyone” (P9) (see Table 2, 4.1a).

#### Men wanted more information about the diagnosis process

Men suggested the written content of Navigate could be improved by being more structured in a way that guided the patient through the diagnosis journey. Several men shared that they were initially unaware of how invasive the biopsy procedure was: “I didn’t realise the biopsy would be such a big deal . . . in this case it was quite a large experience of being admitted to hospital” (P15). Men expressed that, thinking back, they would have wanted more information to prepare themselves for what to expect when having to go through the biopsy procedure under AS management (see Table 2, 4.2a).

Enhancing the Web site by tailoring information relating to their Gleason score diagnosis was also suggested. Some men with intermediate-risk PC revealed they were primarily confused about their Gleason score 7, where it was “a grey area” between choosing an invasive treatment option and being offered AS as an option: “I’m almost too high for the AS. Because my Gleason is 7. . . . It’s borderline” (P9). Therefore, men advised that there should be a section dedicated to clarifying what a Gleason score 7 means in relation to the treatments offered (see Table 2, 4.2b).

#### Some men thought the Web site was limited in presenting the different treatment options available

Several men were concerned about Navigate’s lack of coverage of newer treatments, where one man thought it was “unbalanced”: “it’s still heavily orientated around treatments that were the same . . . treatments which existed 20 years ago” (P18). However, men recognized that these experimental treatments were not readily available in Australia: “there are some other treatments that aren’t widely accepted in Australia. . . . There is not a lot of information on them . . . because they are not widely practised and there’s not a lot of data history” (P13). Men therefore suggested Navigate should be regularly updated to include the most up-to-date treatments (see Table 2, 4.3a). One man also highlighted the importance of including a clinical trials section on Navigate to promote the latest research on offer to men: “some men are choosing radical prostatectomy because they are unaware . . . or can’t afford other options for treatment, which are from time to time available as a clinical trial” (P18).

### Theme 5: Men Identified Design Flaws in the VCE But Appreciated the Tool Being Available

#### Men had usability issues with the VCE

More than half of the men interviewed did not use the VCE tool on Navigate, or did not remember using it, with the main reason being that they “just didn’t see it” (P4). One man suggested that the “Compare My Options” button that leads to the VCE would have been “more noticeable in the middle” of the Web site menu bar rather than in the corner (P4). While going through the VCE questions during the interview, several men thought the wording of some questions was ambiguous and vague (see Table 2, 5.1a). Most were not aware of the “Background Information” button that described what the question was referring to in more detail. After the interviewer explained this to them, the men indicated that they understood the questions better. As a result, men offered suggestions on how to improve the visibility of the Background Information button, such as changing the color and enlarging the font to make it stand out (see Table 2, 5.1b) or altering the wording of the prompt to “Click here for more info” (P2).

#### VCE responses were mixed

The few men who used the VCE at time of diagnosis revealed they used it after they already made their decision, where it was valuable in confirming that they had made the right choice: “it is a good tool to reaffirm my decision” (P13) (see Table 2, 5.2a). One man attempted the VCE multiple times, but the final result did not reflect his chosen treatment (see Table 2, 5.2b). A different man mentioned that he did not want to use it when he was diagnosed because he had already made a treatment decision, and he “didn’t want to confuse [himself] again if it came up with something different” (P10).

#### Men’s overall impressions of the VCE were positive

Men generally appreciated the VCE tool being available and understood its purpose of helping them clarify their treatment decision: “That is a very effective decision-making tool, because it just rules some things in and some things out” (P9) (see Table 2, 5.3a). Men expressed that they thought the VCE questions were confrontational but acknowledged that it was necessary to inform them of what the possibilities are: “It opens your eyes a bit that things like that could happen, it’s a little bit scary” (P6) (see Table 2, 5.3b).

#### Men’s recommendations for future patients

Most men (90%) agreed they would recommend the Navigate Web site to another patient to assist them through their treatment decision-making process. They emphasized that it covered all the different aspects relevant to help men make an informed decision about their treatment, where 1 participant proclaimed that it “should not just be recommended but made mandatory” (P9). Two men did not recommend the Navigate Web site, however, with one reason being that it had “insufficient information about the importance about what a man can do before diagnosis, after diagnosis, before treatment and after treatment” (P18), and another man thought the Web site was “duplicating” other PC resources that were already available (P20).

#### Introducing the Navigate Web site

Men who recommended Navigate suggested it should be introduced at the time of diagnosis. Around half of the men suggested that the specialist should introduce Navigate to their patient since they are the expert who is diagnosing them of the PC and the Web site would be an important resource in prompting questions to ask before consultations. One man cautioned that the specialist’s treatment recommendation may bias men’s decision before considering all their options: “Some people might say there’s no reason to use the Navigate Web site after you’ve seen the specialist” (P4). Several men also advised that it should be the general practitioner (GP) who informs their patient of the Web site before seeing their specialist, to give them an early insight into available options and a better understanding of the terminology. This was particularly important for men who lived in rural areas who saw their local doctor more frequently than their specialist.

## Discussion

This study provides in-depth insight into men’s experiences with LPC diagnosis and their thoughts and experiences using the Navigate DA Web site. Throughout the interviews, it was learned that clinicians had a major role in influencing men’s decision making. In response, the interview schedule was modified throughout the interview period to query men’s interactions with clinicians. The interviews highlighted that men’s treatment decision making was greatly influenced by the perceived authority and trust in their clinician,^26,27^ which led to men deciding on a treatment that aligned with clinician preferences during their consultation. Since these specialist consultations occurred before being introduced to the Navigate Web site, this affected the use of Navigate as a decision-making tool. Clinicians therefore have an important role in explicitly promoting DAs for men to use before and during the consultation.^28^ Improved uptake of DA usage may include e-mail or telephone reminders to patients to use the DA before their consultation^29^ and for clinicians to overtly encourage men to share their preferences and engage in shared decision making during the consultation.^28^

Although Navigate was not the primary influence on men’s treatment selection process, it was evident that men considered it helpful in supporting their ongoing treatment decision making. Written articles and the treatment comparison table were useful in offering detailed and reliable information about their selected treatment that other Internet resources did not provide. Despite the Internet being one of the most common ways for older people to search for health-related information,^30,31^ men expressed apprehensions about the quality of information they accessed on the Internet. The Navigate Web site was able to address these concerns, where men were particularly satisfied that all the relevant information was conveniently placed in 1 Web site that was easy to use.

Other than clinician recommendations, anecdotal experiences from friends and support from partners were also identified as playing a role in men’s decision making, in line with previous research.^32^ The videos of patient process and experience narratives on the Navigate Web site were also considered highly valuable in helping men come to terms with their own diagnosis and prepared them for what to expect with their chosen treatment. A previous review similarly emphasized that unbiased narratives in DAs may have a positive impact on engaging, enlightening, and comforting patients when having to make a decision about their situation,^33^ highlighting video narratives as a key design feature of Navigate and DAs more broadly.

While generally highly satisfied with the Navigate Web site, men also identified and discussed various areas they thought could be improved. Most notably was the suggestion to include more structured written information that could guide men through their prostate cancer diagnosis journey, particularly with the biopsy procedure and understanding their Gleason score. Properly informing men of what to expect regarding a prostate biopsy may be important to ensure adherence to AS management, which requires regular biopsy procedures to monitor disease progression.^34^ Furthermore, men’s understanding of the Gleason score system has been found to be suboptimal.^35^ Thus, it is recommended to address this more prominently on the Navigate Web site.

While Navigate lists creators and includes endorsements from national cancer organizations and urology specialists, men suggested these credentials need to be more prominent to gain the trust and improve engagement from users.^36^ Men indicated they valued knowing the sources of statistical data, particularly in the treatment comparison table, to ensure to users that the information is reliable. To improve trustworthiness in the Navigate Web site, clinicians could be acknowledged as authors on the Web pages in which they could publish updated guidelines and treatments for PC patients.^37^ In addition, inserting a visual cue of an image of Australia on the Web site may remind patients that the Web site is tailored specifically for their needs.

The interviews indicated that half of the men did not use the VCE that was intended to help men deliberate on choosing a treatment based on their preferences and values. Consequently, interview questions were then adapted to investigate the reasons why men did not use the VCE and recommendations to improve the VCE design. This was largely due to design flaws identified in which men indicated that they did not notice the VCE button on the Web site. However, men’s reflections on the VCE during the interview indicated that they understood the aim of using it and thought it was a highly valuable tool. This suggests that the VCE has the potential to help men in the decision-making process, but design adjustments need to be made to improve visibility to users. Men also recommended clearer instructions for the VCE and to cater to users who are less competent with technology, particularly locating the tool. This insight emphasizes the importance of optimizing DA users’ experience by acknowledging age-related issues of cancer patients, including cognitive and functional decline.^38^

### Limitations and Recommendations

Despite the Navigate RCT specifically recruiting men with LPC who were yet to make a treatment decision, many already had an initial treatment in mind prior to using the Navigate Web site. Therefore, while it was created as a decision-making tool, men instead tended to use Navigate as an information tool to better inform themselves and confirm their initial treatment decision. The generalizability of the findings is also limited to predominately privately insured patients, who perhaps had more flexibility in finding and choosing a specialist who could provide a treatment that aligned with their preferences. Thus, the findings may not reflect the experiences of men recently diagnosed with LPC and men who undertake their treatment through the public health system. A further limitation is that the interview data are subject to recall bias, whereby men’s current thoughts about their treatment decision-making experience may not be an accurate depiction of their initial experiences at the time when they were diagnosed. Lastly, given few men chose invasive treatments compared with AS, it was not possible to conduct a subanalysis to examine similarities and differences between treatment types. Further investigation of whether specific themes are more salient among men who choose a particular treatment type would be ideal. Nevertheless, several design and content modifications were identified to improve the Navigate Web site for future implementation in routine clinical practice. Previous research indicates that clinicians play an important role in facilitating the use of PC DAs among patients.^39,40^ Considering the main barrier that led men to use the Navigate Web site as an information resource instead of a decision-making tool was timing, optimization would require that Navigate be made available at diagnosis while men are undergoing treatment decision making with their clinician. Future research could focus on gaining insight into specialists’ perceptions of the Navigate Web site and how to successfully implement it in their clinical practice. It may also be worth considering the influence GPs have in encouraging men to use the Navigate Web site since they typically already have an established relationship with patients.^41^

## Conclusion

Overall, this study highlights how men used the Navigate Web site as an information source to improve their understanding of their chosen treatment. Men appreciated the simple Web design and content that was available in different formats on the Web site. Men also highlighted areas of improvement, including promoting the Web site as being endorsed by reputable experts and showing data sources more prominently; incorporating more written information about the diagnosis process and latest treatments; using visual cues to highlight the relevance of the information; and tweaking design elements of the VCE to make it more user friendly. Most notably, specialist authority appeared influential in guiding men to make an early treatment decision. Therefore, it is recommended that future research focus on exploring optimal timing of introducing DAs and the clinician’s role in encouraging men to use the Navigate Web site to make an informed decision based on their values and preferences.

## Supplemental Material

sj-docx-1-mpp-10.1177_23814683231198003 – Supplemental material for A Qualitative Study of Men’s Experiences Using Navigate: A Localized Prostate Cancer Treatment Decision Aid Click here for additional data file. Supplemental material, sj-docx-1-mpp-10.1177_23814683231198003 for A Qualitative Study of Men’s Experiences Using Navigate: A Localized Prostate Cancer Treatment Decision Aid by Elizabeth Todio, Penelope Schofield and Jessica Sharp in MDM Policy & Practice
