# Supplementary material for: A Qualitative Study of Men’s Experiences Using Navigate: A Localized Prostate Cancer Treatment Decision Aid
Source: MDM Policy Pract. 2023 Sep 13;8(2):23814683231198003. doi: 10.1177/23814683231198003 (PMC10501076; doi:10.1177/23814683231198003)
Supplement: sj-docx-1-mpp-10.1177_23814683231198003 – Supplemental material for A Qualitative Study of Men’s Experiences Using Navigate: A Localized Prostate Cancer Treatment Decision Aid [file sj-docx-1-mpp-10.1177_23814683231198003.docx]

**Supplementary material**

**Supplement A – Navigate website: Homepage**

**
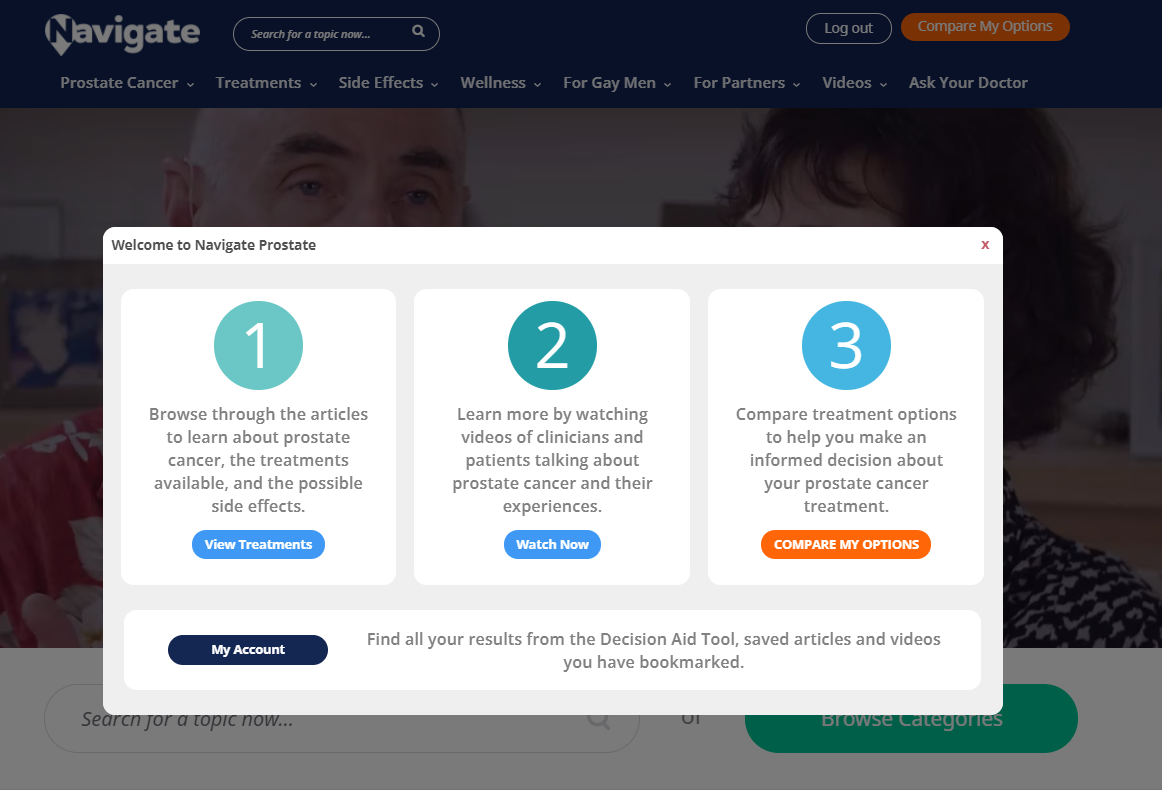
**

**
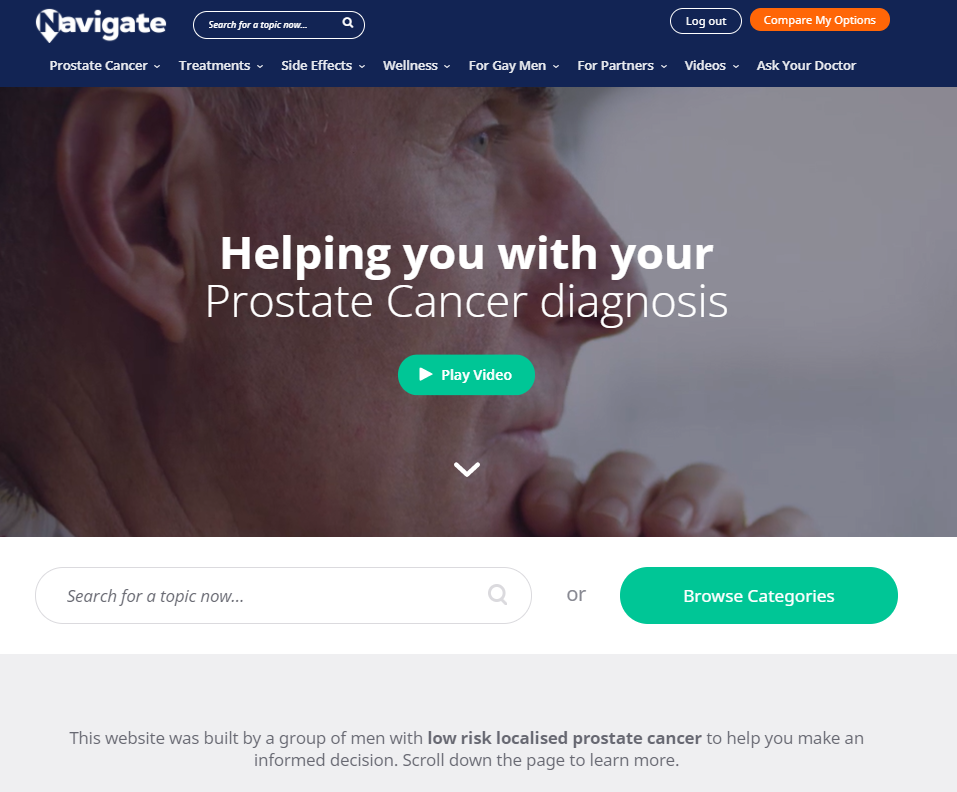
**

**Supplement B – Navigate website: Treatment Comparison Table**


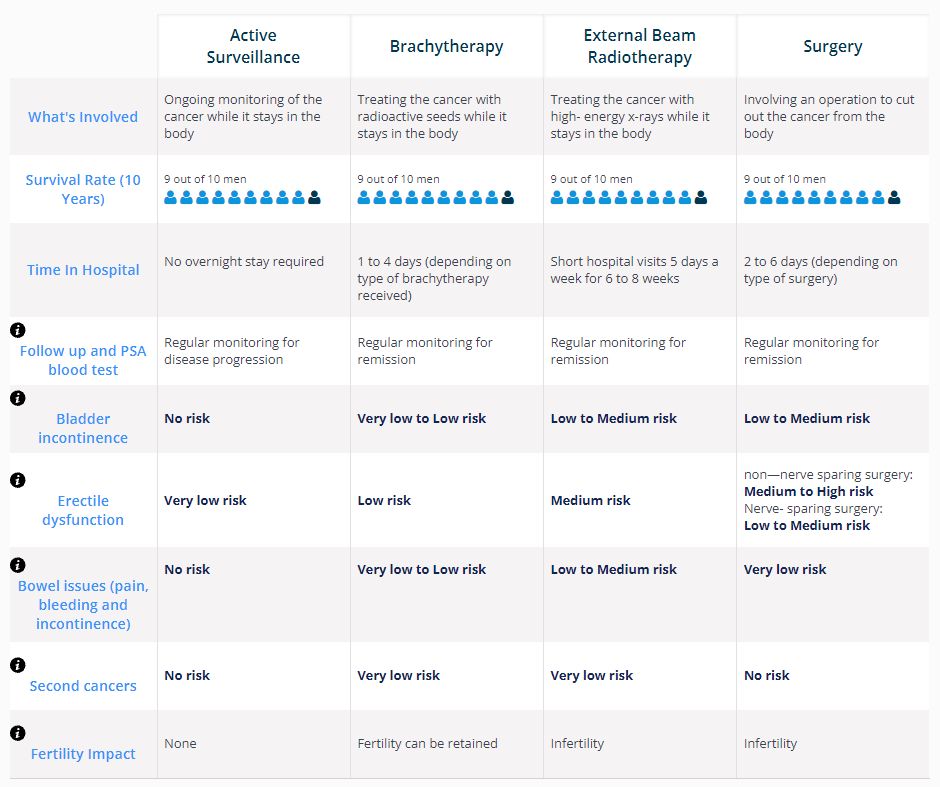


**Supplement C – Navigate website: Values Clarification Exercise**

**
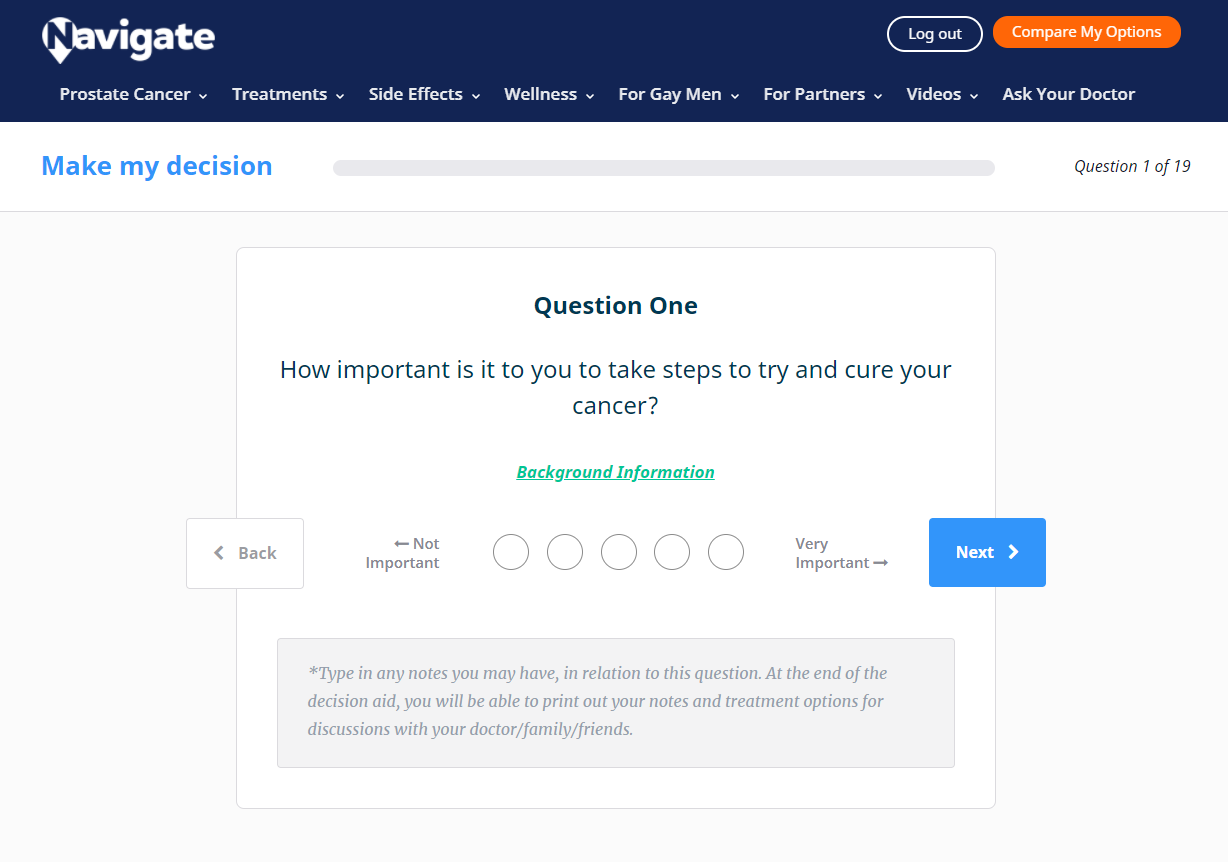
**

**Supplement D – Interview schedule**

Thank you so much for meeting with me today. So as you know, I am the researcher of the Navigate website interview study. Where I will be asking you questions about your experiences using the Navigate website. Do you have any questions about this study? << answer questions>>. Just a couple things before we begin. First, I want to remind you that this interview will be recorded, but when transcribed all identifying information will be removed. Secondly, I’m here to learn from you, so anything you have to share is welcome. There are no right or wrong answers to any of today’s questions. We can also stop the interview at any time and you can withdraw your consent at any time if you wish. This interview will take around 45 minutes to 1 hour of your time. So now I am going to ask you for your consent to participate and start the recording now. <<< start the recording>>> *[Name]* do you understand the information about this study and agree to participate in this interview?

*[Patient verbally agrees]*

Do you have any questions before we begin?

**Demographics**

**Just so I can learn a little bit more about you, I’m going to ask you some basic demographic questions.**

1. What is your age?
2. Do you currently have a partner?
3. What state do you live in?
4. Do you live in a Metro or Regional/Rural area?
5. Are you a public or private patient?

**Relating to treatment choice**

**So before we start talking about the Navigate website, I’d like to ask you a few questions about your experiences from when you were first diagnosed.**

1. Firstly, can you tell me what treatment option you have previously and/or have currently chosen for your diagnosis?
2. How confident or prepared were you in making a treatment decision?
3. How satisfied were you with your treatment decision?
4. Can you tell me about the different types of treatment options that were available for you to choose when you were first diagnosed? Where did you find out about those treatments?
5. How have your thoughts changed (if at all) about choosing your treatment option?
   1. If treatment changes: Can you tell me about your experiences choosing the treatment option of ___? Why did you change your decision?
6. Before you had access to the Navigate website, can you tell me about the kinds of resources that were available to you to help you make a decision about your treatment? Probe for details (eg information, discussions, doctors).

**Initial Response**

**Now I’d like you to think about the time that you were first given access to the Navigate website, which would have been up to three months after your initial diagnosis**

1. When you first logged in, what were your initial impressions of the website?
2. Other than looking at the website recently before this interview, did you end up revisiting the website? Why?

We will now briefly go through the Navigate website to give you a refresher of what the different areas of the website are.

*[While showing the patient the Navigate website]* So on the website there are different articles that you can browse through explaining your diagnosis, the treatments available, and the possible side-effects etc. In addition, there are videos of doctors, prostate cancer patients and their partners explaining their experiences. Lastly, there is the decision aid tool, which is also known as the ‘Compare my options’ button, that contains 19 questions that ask you about your values and preferences in relation to your diagnosis to help you decide on your treatment option. Once you complete the questionnaire, this table appears that shows you the two most appropriate treatment options based on your answers. Do you have any questions or comments so far?

**Design and usability**

**Now I’m going to ask you questions relating to the look of the website:**

1. What are your thoughts about the ‘look’ and ‘feel’ of the website, Further prompts: the colours; the size of the writing; anything about the way it is presented?
2. Do you have suggestions on how to improve the design of this website? If yes, use further prompts
3. What are your thoughts about how to access the information you wanted on the website?
4. How easy or difficult was it to move through the different areas of the website?

**Articles and Videos**

**Now we’re going to look at the articles and videos on the website:**

1. What did you think about the written articles on the website? (Probe for details e.g. easy to read?, language pitched at right level, images, resources provided, level of detail, interesting and engaging)
2. Which ones did you access/read through? Or recall?
3. What did you think about the videos on the website? (Probe details)
4. Which ones did you access/read through? Or recall?

**Decision-aid**

**Now we’re going to look at the decision aid, which is the ‘Compare my options’ button:**

1. Did you click on the ‘Compare my options’ button when using the website? What are your thoughts on the phrase ‘Compare my options’? Did you understand what it meant?
   1. Yes:
      - What did you think about the tool?
      - How did you find using the tool?
      - How useful was the decision-aid tool in helping you select a treatment option?
      - How clear were the questions in the DA?
      - Did the questions make sense to you?
      - How often did you use the DA tool? (More than once: What made you use the DA tool more than once?)
      - Do you have any suggestions on how to improve the DA tool?
   2. No:

- Why did you not use the decision-aid?
- Can you think of anything that could have made you more likely to use the decision-aid?
- I know you said that you didn’t use the DA tool, but do you have any suggestions on how to improve the DA tool, just based on looking through it now?

**Post-website experiences**

**Now I’m going to ask you questions relating to your overall thoughts about the website:**

1. What was your overall impressions of the website? Can you please elaborate?
2. Were there any areas of the website that were particularly helpful to you? What was the best part?
3. Were there any areas that were not as helpful or confusing? How can this be improved?
4. Were there any topics that you feel were not covered on the website that should be included?
   1. If yes: Please tell me what you think should be added.
5. Were there any topics that you think should not have been included on the website?
   1. If yes: Please tell me what you think should be excluded.
6. Did the website have any effect on your views about the treatment you were considering or leaning towards?

a. If yes, in what way?

b. If no, why do you think it didn’t have an effect?

**Recommendations for future patients**

**These last few questions will ask about your thoughts and recommendations of this website to future patients:**

1. On reflection, would you recommend this website to a man trying to decide on the best treatment for them?
2. When do you think it would be the best time to make it available to patients?
3. Who should introduce it (GP, Urologist, Prostate Cancer Nurse)?

I have no further questions. But before we wrap up, is there anything else you would like to bring up, or ask about, before we finish the interview?

That brings this interview to a close, I really appreciate you taking the time to share your insights and for participating. We anticipate that the study results will be available in 2022. Would you like to receive a copy?

CLOSE INTERVIEW
